# A simulation-based breeding design that uses whole-genome prediction in tomato

**DOI:** 10.1038/srep19454

**Published:** 2016-01-20

**Authors:** Eiji Yamamoto, Hiroshi Matsunaga, Akio Onogi, Hiromi Kajiya-Kanegae, Mai Minamikawa, Akinori Suzuki, Kenta Shirasawa, Hideki Hirakawa, Tsukasa Nunome, Hirotaka Yamaguchi, Koji Miyatake, Akio Ohyama, Hiroyoshi Iwata, Hiroyuki Fukuoka

**Affiliations:** 1 NARO Institute of Vegetable and Tea Science (NIVTS), 360 Kusawa, Ano, Tsu, Mie 514-2392, Japan; 2 Department of Agricultural and Environmental Biology, Graduate School of Agricultural and Life Sciences, The University of Tokyo, 1-1-1 Yayoi, Bunkyo-Ku, Tokyo 113-8657, Japan; 3 Kazusa DNA Research Institute, 2-6-7 Kazusa-kamatari, Kisarazu, Chiba 292-0818, Japan; 4 NARO Institute of Vegetable and Tea Science (NIVTS), 3-1-1 Kannondai, Tsukuba, Ibaraki 305-8666, Japan

## Abstract

Efficient plant breeding methods must be developed in order to increase yields and feed a growing world population, as well as to meet the demands of consumers with diverse preferences who require high-quality foods. We propose a strategy that integrates breeding simulations and phenotype prediction models using genomic information. The validity of this strategy was evaluated by the simultaneous genetic improvement of the yield and flavour of the tomato (*Solanum lycopersicum*), as an example. Reliable phenotype prediction models for the simulation were constructed from actual genotype and phenotype data. Our simulation predicted that selection for both yield and flavour would eventually result in morphological changes that would increase the total plant biomass and decrease the light extinction coefficient, an essential requirement for these improvements. This simulation-based genome-assisted approach to breeding will help to optimise plant breeding, not only in the tomato but also in other important agricultural crops.

Genetic improvement of plant species, generally known as breeding, has played an important role in the development of human societies^1^. In order to secure a stable food supply, farmers had to improve yield and plant resistance to biotic and/or abiotic stress. As a well-known example, the breeding of high-yielding semi-dwarf varieties of wheat and rice greatly contributed to the Green Revolution, which significantly increased food production and helped avoid a chronic food shortage after the rapid increase in world population in the 1960s^2^. High-nutrient crops are an important part of the solution to the world problem of ‘hidden hunger’^3^, or micronutrient deficiency. In addition, economic and social development have generated new demands for food plants that provide good flavour and nutrition for optimal health^4^. So far, plant breeding has mainly involved cycles of crossing and selection that require a long time commitment and great effort from plant breeders. However, it is necessary to develop more efficient breeding methods in order to increase yields and feed a growing world population^5^, as well as to meet the demands of consumers who have diverse preferences and require high food quality^4^.

It is widely accepted that genomic information will be used to develop efficient breeding systems^6^,^7^. Remarkable advances in next-generation sequencing technology have made abundant low-cost molecular markers available^8^. This development has allowed the aggressive use of molecular markers for plant breeding. Genome-assisted breeding may be classified roughly into two categories, the first of which includes marker-assisted selection (MAS)^9^ and marker-assisted recurrent selection (MARS)^10^, while the second is genomic selection (GS)^11^,^12^,^13^,^14^. With MAS and MARS, information on the genotype of markers that are significantly associated with phenotypic variation is used as an indicator of introgression. Thus, a breeder can confirm the introduction of new genes or quantitative trait loci (QTLs) without observing phenotypic variation of target traits.

Unlike MAS and MARS, GS does not focus on the association between phenotypic variation and the genotype of any specified markers. In GS, selection candidates are chosen on the basis of predicted genetic potential (i.e. genomic estimated breeding values, GEBVs) calculated by whole-genome prediction (WGP) models. The WGP model is designed to predict GEBVs by using genome-wide DNA markers as explanatory variables. GS was first proposed by Meuwissen *et al.*^15^ and its efficiency was demonstrated in the breeding of dairy cattle^16^. GS outperforms MAS and MARS, especially when the target trait is controlled by a large number of QTLs. Because most agronomically and economically important traits are controlled by a number of QTLs with small to medium effects and it is difficult to improve them using MAS, GS has begun to attract attention from breeders and geneticists.

Although GS has significantly contributed to animal breeding, it is difficult to apply a strategy that is efficient in animal breeding directly to plant breeding, due to the differences in breeding situations. Most of the literature related to GS in plants focuses on evaluating prediction models^17^. Furthermore, a theoretical method that will describe how to apply GS to actual plant breeding schemes is needed. For example, in the development of plant varieties, two or more traits are often the targets of improvement. Even when only one trait is a target, it is necessary to evaluate the genetic potential of multiple traits that are agronomically important. However, few studies have addressed this need.

In order to apply GS to a plant breeding programme in which changes in multiple traits are important, we propose a strategy that integrates a computer simulation of the breeding process and an evaluation of the simulated breeding population by using WGP models. In this study, for the proof of concept of this simulation-based method, we used simultaneous improvement of yield and flavour in tomato (*Solanum lycopersicum*) as an example. Our simulation indicated that it is necessary to perform cycles of crossing and selection to simultaneously improve yield and flavour, confirming that, as suggested in previous studies, this is a difficult breeding objective^18^,^19^,^20^,^21^. In addition, our simulation predicts that simultaneous breeding selection for yield and flavour eventually results in the morphological changes that have been proposed as essential requirements for these improvements in previous physiological studies. Throughout the present study, we demonstrate that a simulation-based method with highly accurate WGP models enables not only estimation of genetic gains regarding target traits but also prediction of the influence of the selection on non-target traits.

## Results

### Phenotypes

In the present study, 96 big-fruited tomato varieties (Supplementary Table S1) were phenotyped for 20 agronomic traits (Table 1; Supplementary Fig. S1). One plant of each variety was grown each year, for four years. The phenotypic values were averaged over the years (Supplementary Note). Traits categorised as ‘quality’ relate to commercial value due to consumer preferences. Traits categorised as ‘physiological disorder of fruit’ are important (and undesirable) in tomato production because they represent a reduction in the marketable fruit yield and the farmer’s profit. Traits categorised as ‘others’ are general agronomic traits that are directly or indirectly associated with yield and quality traits. Among the traits that were analysed, pericarp colour was the only qualitative trait (Supplementary Fig. S1). The broad-sense heritability of the traits ranged from 0.10 to 1.00 (Table 1). These relatively high levels of heritability are probably due to the high stability of plant growth under hydroponic cultivation (see Methods).

### Linkage disequilibrium and population structure analysis

**Figure 1:**
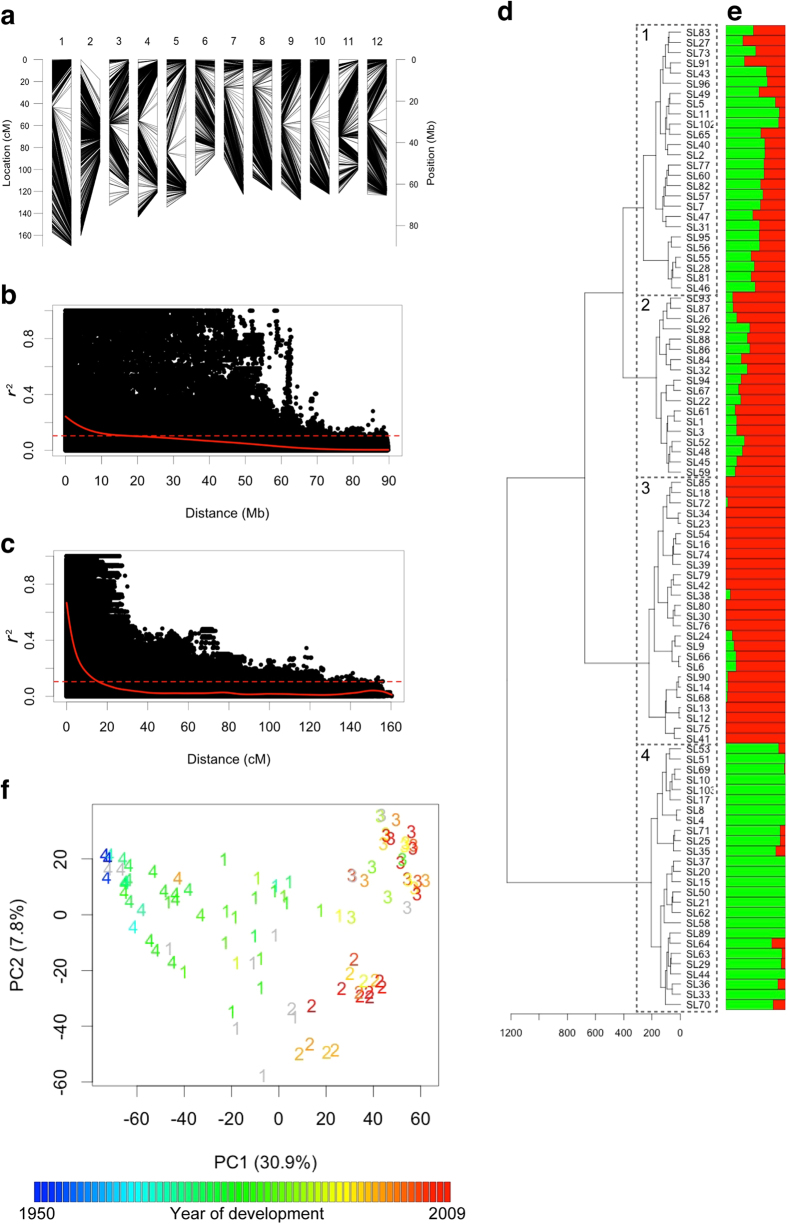
Linkage disequilibrium (LD) and population structure analysis. (**a**) Linkage and physical map positions of SNP markers used in the present study. The numbers at the top of the panel indicate chromosome number. Lines indicate the chromosomal distribution of SNP markers. The left and right sides of each line indicate the linkage and physical map positions, respectively. (**b**) Plot of LD values (*r*^2^) against physical distance. The curve indicates local polynomial fits using kernel smoothing regression. The horizontal dashed lines represent the baseline *r*^2^ values based on the 95th percentile of the distribution of *r*^2^ values between pairs of unlinked markers. (**c**) Plot of LD values (*r*^2^) against linkage map distance. (**d**) Hierarchical clustering using the Ward method. (**e**) Bayesian clustering. Each variety was divided into two hypothetical subpopulations based on the population membership coefficients. Each subpopulation is represented by a different colour. (**f**) Principal component analysis. The numbers in plots correspond to the clusters in the hierarchical clustering. The colour of each plot indicates the year of development of each variety.

In tomato, several high-density single-nucleotide polymorphism (SNP) marker sets have been developed, and their usefulness has been verified in diversity panels that include a wide range of varieties and wild relatives^22^,^23^,^24^,^25^. However, these marker sets were insufficient to capture the genome-wide SNP variation in the varieties used in the present study. Specifically, there were several genomic regions for which no polymorphic markers were available. This lack of data is probably due to the bias in the genetic variation used in the present study, namely, only big-fruited commercial varieties were used in our study, whereas diversity panels were used in previous studies. The bias may have complex effects on measures of diversity and relationships among varieties^26^. Therefore, we developed new SNP marker sets to conduct the genetic analysis of varieties in the present study (see Supplementary Methods). A total of 16,782 SNPs with minor allele frequency >0.05 were selected (Fig. 1a).

The tomato genome has large pericentromeric regions^27^, which makes it difficult to interpret the extent of linkage disequilibrium (LD). Therefore, we estimated the linkage map position of each SNP from the physical position by using the linkage map information in Shirasawa *et al.*^28^. The average marker interval of SNP markers in the present study was 0.09 cM according to linkage map positions (Fig. 1a). LD is closely associated with the performance of WGP. In the varieties used in the present study, the degree of LD was estimated to be 10–20 Mb and 10–20 cM (Fig. 1b,c). Muir^29^ indicated that when markers and QTLs are in linkage equilibrium, the accuracy of WGP decreases, due to the difficulty of capturing the effects of QTLs using SNPs. Because the average marker interval in the present study is far smaller than the estimated LD, the density of the marker set in the present study is sufficient for WGP.

As a highly structured population is not suitable for genetic analysis using WGP^30^, it was necessary to perform a population structure analysis in order to estimate the efficiency of WGP. The population structure of the varieties was estimated via hierarchical clustering, Bayesian clustering, and principal component analysis (PCA). In hierarchical clustering, the 96 varieties were divided into four major clusters (Fig. 1d). In Bayesian clustering, the optimal number of subpopulations was estimated as *K* = 2 according to the value of ∆*K*^31^ (Fig. 1e). On the basis of the results from the Bayesian clustering, clusters 3 and 4 in hierarchical clustering were composed of different subpopulations, whereas clusters 1 and 2 were composed of populations that were intermediate between clusters 3 and 4 (Fig. 1d,e). Both Bayesian clustering and PCA indicated that the varieties in this study did not represent a highly structured population and could therefore be used for WGP (Fig. 1e,f). Most of the varieties in clusters 1 and 4 were developed before 1990, whereas most of the varieties in clusters 2 and 3 were developed after 2000 (Supplementary Table S1). The relationship between the population structure and the year of development was more evident in PCA (Fig. 1f). PC1, which explains 30.9% of genetic variation, was highly correlated with the year of development of each variety (*r* = 0.82). Thus, a population structure analysis revealed the relationship between the change in genome composition and the history of tomato breeding.

### Genome-wide association study

A genome-wide association study (GWAS) that detects the association between polymorphic patterns of DNA markers and phenotype values is now a common strategy to detect QTLs that are available for MAS or MARS^32^. Prior to the assessment of WGP, we performed GWAS using two statistical methods: mixed linear model (MLM)^33^ and extended Bayesian Lasso (EBL)^34^. MLM is a standard method that analyses the association between a single marker and phenotypic values by using a genetic relationship matrix and other optional parameters as covariates. EBL is a Bayesian shrinkage method that analyses the significant association of all markers with phenotypic values simultaneously.

**Figure 2:**
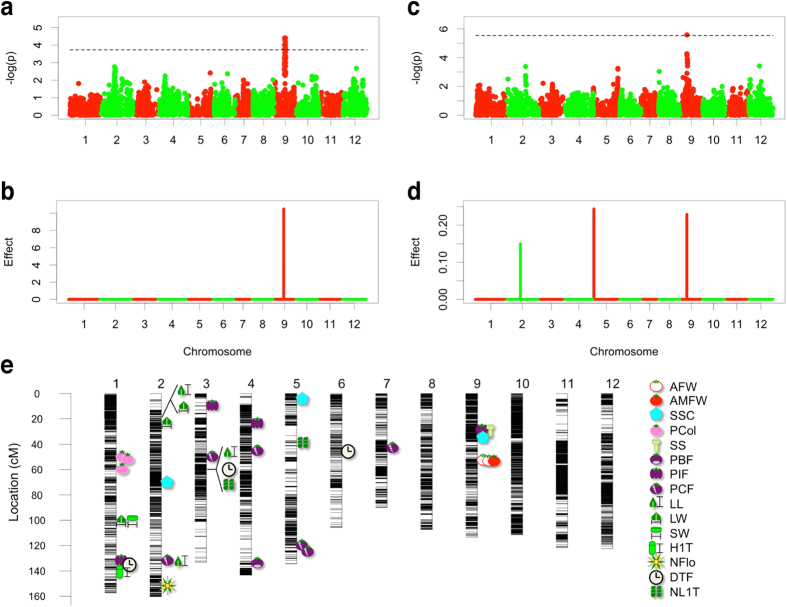
Summary of the genome-wide association study (GWAS) results. (**a,b**) GWAS results for average marketable fruit weight. (**c,d**) GWAS results for soluble solids content. (**a,c**) Manhattan plots for mixed linear model. The horizontal dashed lines indicate the threshold obtained from the 5% false discovery rate. (**b,d**) Posterior means of all marker effects for extended Bayesian Lasso. The values were obtained by using hyperparameter *θ* = 0.0001. (**e**) Chromosomal distribution of significant associations detected by the GWAS. AFW, average fruit weight; AMFW, average marketable fruit weight; SSC, soluble solids content; PCol, pericarp colour; SS, style scar; PBF, percentage of blossom-end rot fruits; PIF, percentage of irregular-shaped fruits; PCF, percentage of cracked fruits; LL, leaf length; LW, leaf width; SW, stem width; H1T, height to the first truss; NFlo, number of flowers; DTF, days to flowering; NL1T, number of leaves under the first truss. See Table 1 for details.

Using the GWAS, MLM and EBL detected 4 and 34 putative QTLs, respectively (Supplementary Table S2). Among the QTLs, 3 were common to both methods, and 35 QTLs were detected in all (Fig. 2). The positions of several significant associations corresponded to genes or QTLs that have been identified in previous studies. A significant association between AX-107553846 (5015774 bp on chromosome 9) and average marketable fruit weight was located on the region of *fw9.1*, originally identified as a QTL between cultivated and wild tomato (Fig. 2a,b; Supplementary Table S2)^35^. AX-95792472 (2623609 bp on chromosome 9), which is associated with soluble solids content, was located close to *Lycopersicum Invertase 5*, which controls soluble solids content in fruits (Fig. 2c,d; Supplementary Table S2)^36^,^37^. In addition, AX-95802300 (71269940 bp on chromosome 1), which is associated with pericarp colour, was located close to *SlMYB12*, which is known to control the accumulation of flavonoids on the fruit surface (Fig. 2e; Supplementary Table S2)^38^,^39^.

In order to assess MAS and MARS using the significant associations in the GWAS, we investigated the predictability of the linear regression model using significant associations as explanatory variables (Table 2). It is noteworthy that the predictability of yield-related traits, such as average fruit weight, was very low. Regarding total fruit weight, no significant association was detected (Fig. 2; Supplementary Table S2). These results suggest that the genetic gains obtained via MAS and MARS are very small for these traits.

### Evaluation of WGP models

For WGP, we used five linear methods, including Ridge regression (RR)^40^, Bayesian Lasso (BL)^41^, EBL^34^, weighted Bayesian shrinkage regression (wBSR)^42^, and Bayes C^43^, as well as two nonlinear methods, reproducing kernel Hilbert space regression (RKHS)^44^ and random forest (RF)^45^. The accuracy of the prediction was evaluated by correlation coefficients between GEBVs and phenotypic values via leave-one-out cross-validation (LOOCV) analysis (Table 3). For most of the traits, WGP seemed to show higher accuracy than multilinear regression models, according to the GWAS results (Tables 2 and 3). In particular, regression models with significant markers in the GWAS showed low accuracy for the prediction of traits related to yield (Table 2). WGP is obviously a better genome-assisted breeding strategy than MAS and MARS for these traits. High prediction accuracy was also observed for soluble solids content, which is closely related to flavour in tomato fruits^4^ (Table 3). Thus, LOOCV analysis revealed the high potential of WGP to improve important traits in tomato.

### Simulation of recurrent genomic selection

Even if WGP is highly accurate, its use in breeding is hindered by the problem of whether it is possible to develop populations or lines that possess high GEBV through realistic crossing and selection. To address this issue, it is helpful to predict GEBVs for future crosses^46^,^47^. Therefore, to assess the use of WGP in tomato breeding, we predicted the segregation of GEBVs in future crosses. We used the Poisson distribution for the number of recombination, under the assumption that the length of each chromosome in the Morgan’s unit in the linkage map is the lambda parameter^28^. This approach enabled precise reproduction of genetic recombination and simulation for generations of genomic change.

**Figure 3:**
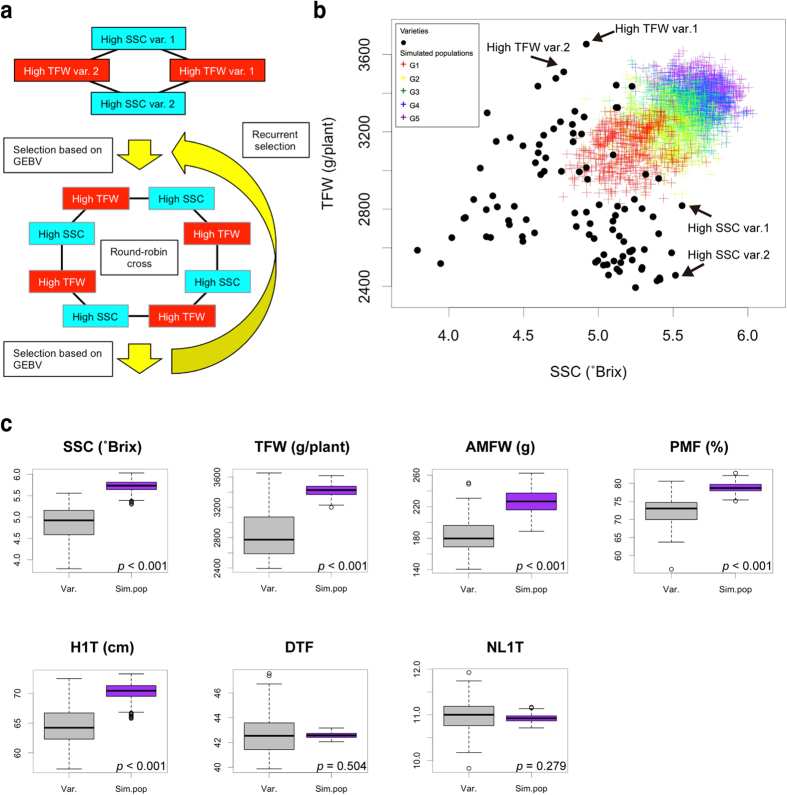
Results of simulations for the recurrent genomic selection. (**a**) Scheme of breeding strategy used in the present study. (**b**) Distributions of the genomic estimated breeding values (GEBVs) of total fruit weight (TFW) and soluble solids content (SSC). Black circles and coloured crosses indicate the GEBVs of the 96 varieties and the simulated populations, respectively. G1 to G5 indicate the generation of breeding population during the cycles of recurrent genomic selection. (**c**) Boxplots for the GEBVs in the fifth generation of the simulated population (G5 in Fig. 3b). Statistical analysis was performed using Welch’s *t*-test. ‘Var.’ and ‘Sim.pop.’ at the bottom of each panel indicate the 96 varieties and the simulated population, respectively. AMFW, average marketable fruit weight; PMF, percentage of marketable fruits; H1T, height to the first truss; DTF, days to flowering; NL1T, number of leaves under the first truss. See Table 1 for details. GEBVs of each trait were estimated by using the statistical method that showed the highest predictability in leave-one-out cross-validation in Table 3.

The improvement of both yield and flavour is one of the most important breeding objectives in tomato^18^,^19^. Of the traits analysed in the present study, total fruit weight and soluble solids content are most closely associated with yield and flavour performance, respectively. Therefore, we simulated the breeding selection for both total fruit weight and soluble solids content. GEBVs of each trait were calculated using the statistical method that showed highest predictability in LOOCV: Bayes C and EBL were used for total fruit weight and soluble solids content, respectively (Table 3). As an example of this proof-of-concept study, we simulated a breeding strategy that uses a few individuals with high GEBV for each trait as parents (Fig. 3a). Specifically, the first generation was developed by a round-robin cross of the top two varieties in GEBV for total fruit weight and the top two in GEBV for soluble solids content. Later generations were developed by a round-robin cross of the top four progeny in GEBV for total fruit weight and the top four in GEBV for soluble solids content. The population size was kept constant at *n* = 96. The number of progenies from each cross in the first generation was 24 (96/4 crosses), whereas the number was 12 (96/8 crosses) in the subsequent generations (Fig. 3a). Because the result of the simulation is expected to be strongly affected by the genome composition of the selected individuals, we performed five independent simulations.

In the first generation, trait distributions were almost intermediate between the parents, suggesting that recurrent cycles of crossing and selection are necessary to simultaneously improve these traits (G1 in Fig. 3b; Supplementary Fig. S2). The GEBVs increased as the cycles of recurrent genomic selection increased, and in the fifth generation, the GEBVs reached values that were comparable with the varieties possessing the highest value in each trait (G5 in Fig. 3b; Supplementary Fig. S2). Thus, the simulation indicated the need for recurrent genomic selection to improve both total fruit yield and soluble solids content. This result is reasonable because previous studies have recognised the difficulty of simultaneously improving these traits^18^,^19^,^20^,^21^. We also simulated the production of inbred lines derived from each generation in recurrent genomic selection and eventually found that the distribution of GEBVs was similar to that of each of the parental generations (Supplementary Fig. S2b). Because recurrent selection based on phenotypic observation requires an enormous amount of time and labour^48^, WGP-based recurrent selection is a promising strategy to achieve this breeding objective.

During plant breeding, it is also important to investigate the effects of a breeding selection on non-target traits. Therefore, we investigated GEBVs of non-target traits as well as of the target traits (i.e. total fruit yield and soluble solids content) in the simulated population (Fig. 3c; Supplementary Fig. S3). In the simulated population, an increase in average fruit weight was predicted (Fig. 3c; Supplementary Fig. S3). Because the average fruit weight is directly associated with total fruit weight, this result is reasonable. Furthermore, an increase in the percentage of marketable fruits was observed, suggesting that this selection will have few negative effects (Fig. 3c).

Interestingly, in the simulated population, several morphological changes were predicted that were necessary to improve both total fruit weight and soluble solids content. An increase in height to the first truss without an increase in the number of days to flowering indicates an increase in total plant biomass (Fig. 3c). Previous studies revealed that an increase in total plant biomass is important for the further increase of yield performance^49^. In addition, an increase in height to the first truss without an increase of the number of leaves under the first truss indicates a decrease in the light extinction coefficient, due to more space between successive leaves (Fig. 3c). In tomato, photosynthesis performance is strongly related to both yield performance and soluble solids content in fruits. A previous physiological study demonstrated that a decrease in light extinction coefficient significantly contributes to photosynthesis and yield performance^50^. Thus, the simulations in the present study confirmed the theory suggested by physiological studies.

## Discussion

One of the main objectives in the present study was to assess the potential of genome-assisted breeding in plant species. We used commercial elite varieties as plant materials, unlike previous studies that used diversity panels to represent the genetic diversity of tomato^51^,^52^,^53^. Because crossing among elite lines is a common strategy in modern plant breeding programmes, our choice of plant materials was suitable for the purpose. Due to the difference in plant materials, however, the results of the GWAS were very different from those of previous studies.

In the present study, no significant association related to average fruit weight was detected on chromosome 2 in the GWAS (Fig. 2; Supplementary Table S2). It is well known that tomato chromosome 2 carries a number of QTLs involved in fruit weight and size, such as *fw2.2*^54^ and *lcn2.1*^55^, and these were detected in the previous GWAS studies^51^,^52^,^53^. A population used in the present study was composed of big-fruited commercial varieties, and it is possible that all varieties share common alleles for these QTLs that increase fruit weight. Alternatively, we found a significant association for average (marketable) fruit weight on chromosome 9 that probably corresponds to *fw9.1* (Fig. 2; Supplementary Table S2)^35^. In plant breeding, the detection of QTLs that have not prevailed in modern elite varieties is more important than the detection of QTLs that have already been used in past and current breeding programmes. Thus, we demonstrated that the GWAS may be used with elite varieties to detect significant associations that are good candidates for future MAS and MARS work.

In the present study, the WGP models showed relatively high predictability (Table 3). Even when high predictability is observed in a WGP model, the genetic gain from GS is often equal to or less than the phenotypic selection on a per-cycle basis.

However, phenotypic selection for tomato can only be performed once per year, during the cropping season, whereas GS can be performed more than twice per year. Thus, the use of GS can shorten the breeding cycle length and increase gains per unit time^48^. In addition, hydroponic cultivation of tomato requires a large amount of space and enormous effort. Considering these factors, GS with highly accurate WGP is an efficient method for the rapid genetic improvement of target traits in tomato.

In tomato, improvement of both yield and flavour is one of the most important breeding objectives and poses a difficult challenge^18^,^19^,^20^,^21^. In the present study, we assessed the potential of WGP to achieve this breeding objective by using simulations and demonstrated that recurrent selection that uses WGP is an efficient strategy (Fig. 3). In general, the reliability of simulation results depends on the accuracy of WGP models. Even when the WGP model is completely accurate, the results may differ from observations in an actual trial because of randomness in the recombination and the genome structure of the selected progeny during recurrent selection.

Nevertheless, the results of our simulations suggested two important facts that can inform the design of future breeding. First, a single crossing and selection is not sufficient to achieve this breeding objective (G1 in Fig. 3b; Supplementary Fig. S2). Multiple cycles of crossing and selection (i.e. recurrent selection) are necessary; this finding confirms that, as suggested by previous studies, it is difficult to improve these traits^18^,^19^,^20^,^21^. Secondly, breeding selection affects other non-target traits (Fig. 3c; Supplementary Fig. S3). In the simulations in the present study, the increase in total plant biomass and the decrease in light extinction coefficient were suggested from the GEBVs of height to the first truss, days to flowering, and number of leaves under the first truss (Fig. 3c). In previous physiological studies, these factors were considered important factors to increase both yield performance and fruit soluble solids content^49^,^50^. The confirmation of this understanding by the simulations suggests that the WGP models and the simulations used in the present study make it possible to predict the influence of breeding selection on other non-target traits.

In addition, we observed an increase in the percentage of marketable fruits, one of the most important traits for the development of tomato varieties (Fig. 3c). This finding indicates that few negative effects, such as an increase in physiological disorders in fruits, are expected as a result of this breeding selection (Fig. 3c; Supplementary Fig. S3). Even if an increased percentage of physiological disorders is predicted, it may be partially prevented by exploiting a MAS approach that uses the significant associations detected in the GWAS (Fig. 2). Thus, in the present study, we demonstrated that, with highly accurate WGP models, simulation-based design can not only estimate genetic gains but also can predict the influence of the selection on other traits. Therefore, it represents an efficient and elaborate breeding design that considers changes in multiple traits, as is often necessary in plant breeding.

The use of computer simulation for breeding design in plants has been discussed^56^. Although we used tomato in this study, the strategy may be applied to plant species with similar breeding systems, such as wheat and rice. As more and more genotyping platforms and software for genetic and genomic analyses become available, computer simulation will play an increasingly important role in the design of future breeding programmes.

## Methods

### Plant materials and growth conditions

We used 96 big-fruited tomato F_1_ varieties intended for the fresh market. These varieties were developed from 1952 to 2009 by various organisations, such as seed companies and the public sector (Supplementary Table S1). Total genomic DNA was isolated from the leaves of a single plant from each variety using a DNeasy Plant Mini Kit (Qiagen, Hilden, Germany). All plants were grown hydroponically with a high-wire system in a greenhouse at the National Agriculture and Food Research Organization, Institute of Vegetable and Tea Science in Tsu, Japan. Plant growth was started in the second week of August and terminated in January, each year from 2011 to 2014. One plant was grown for each variety, each year.

Tomato seeds were sown on a granular soil (Nippi Engei Baido 1; Nihon Hiryo Co., Tokyo, Japan), and 20 days later, seedlings were transplanted onto rockwool slabs. A mixture of Otsuka-A nutrient solution and Otsuka-5 nutrient solution (Otsuka AgriTechno, Tokyo, Japan) was provided to the plants. The electrical conductivity level was adjusted to 0.80, 1.20, 1.60, 2.00, and 2.40 dS·m^−1^ in accordance with plant growth. The plants received 300 ml of water each time they were watered (six times a day, in accordance with plant growth and climate conditions). To promote fruiting, Tomato-tone (including 0.15% 4-chlorophenoxy acetic acid, Ishihara Biosciences, Tokyo, Japan) was diluted 100-fold and sprayed on each truss when the second to fifth flowers were at the flowering stage. The plants were deflorated to limit the maximum number of flowers per truss to six and were pinched above the fourth truss. A total of 20 traits were phenotyped (Table 1). The phenotypic values were averaged over the years to remove the year effect from the phenotypic values, and the average phenotypic values were used in the analysis (Supplementary Data S1). Thus, we ignored genotype by environment effects, and the validity was assessed in Supplementary Note. The broad-sense heritability (*h*^2^) was calculated from the estimates of genetic (σ^2^_G_) and residual (σ^2^_E_) variances derived from the expected mean squares of the analysis of variance to express the genetic effects of traits:


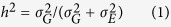


The calculation of σ^2^_G_ and σ^2^_E_ was performed by using the R function ‘aov’.

### Genotype data

We developed a high-density SNP marker set for the genetic analysis of the 96 varieties. The details are described in the Supplementary Methods. In brief, we re-sequenced the 96 varieties with a mean depth of 1.9× by using HiSeq2000 (Illumina Co, Ltd., San Diego, CA, USA), according to the manufacturer’s protocol (DDBJ Sequence Read Archive Submission DRA003755). A total of 51,912 candidate SNPs were selected and genotyped with the 96 cultivars by using Axiom myDesign genotyping arrays (Affymetrix Co, Ltd., Santa Clara, CA, USA) (Supplementary Data S2). From these SNPs, a total of 16,782 SNPs were considered to be effective markers, using the criteria that missing data constituted no more than 5% (*n* = 4) of the variety and that the minor allele frequency was greater than 5% (Supplementary Data S3). The SNP genotype data were provided to BEAGLE version 3.3.2^57^ to impute missing genotype data and estimate the most likely linkage phases of the 96 F_1_ varieties (Supplementary Data S3).

### Linkage disequilibrium

LD between pairs of markers was evaluated by using the function ‘LD’ in the R package *genetics* version 1.3.8.1. The relationship between the degree of LD and the linkage map distance was analysed. The linkage map positions of SNP markers developed in the present study were estimated from their physical positions via local polynomial regression, using the relationship between physical positions and linkage map positions obtained in Shirasawa *et al.*^28^. The local polynomial regression was conducted by using R function ‘loess’ with the default parameter setting, except for the span, which controls the degree of smoothness and was set to 0.189 for this analysis. When the estimated distance between two successive markers became negative, it was replaced with 1.0^−6^. The relation between *r*^2^ values and the linkage map distance between the corresponding markers was modelled by fitting local polynomials with the function ‘locpoly’ in the R package *KernSmooth* version 2.23. A parameter bandwidth for ‘locpoly’ was selected by using the function ‘dpill’ in the R package *KernSmooth*.

### Population structure analysis

The population structure of the 96 varieties was estimated using hierarchical clustering, Bayesian clustering, and PCA. For hierarchical clustering and PCA, SNP genotypes were scored as 0, 2, and 1 for the two homozygotes (e.g. GG, AA) and the heterozygote (e.g. GA), respectively. The hierarchical clustering was conducted based on the Ward method with Euclidean distance by using the R function ‘hclust’. Bayesian clustering was conducted by using the program STRUCTER version 2.3^58^. Markov Chain Monte Carlo (MCMC) cycles were repeated 20,000 times after 10,000 cycles of a burn-in period. In the analysis, we tested the admixed models with the number of populations (*K*) with 1–10. Each test included five independent calculations. Optimal *K* was estimated based on the ∆*K* that is the rate of change in the log probability of data between successive *K* values^31^. ∆*K* was calculated by using STRUCTURE HARVESTER version 0.6.94^59^. Data from the five independent calculations were integrated by using CLUMPP version 1.1.2, which deals with label switching between different calculations that use the same *K*^60^. The *FullSearch* algorithm was used for the estimation. The PCA was conducted by using the R function ‘prcomp’.

### Regression methods

Details for the regression methods used in the present study are described in Supplementary Methods. In the GWAS, we used two regression methods, MLM^33^ and EBL^34^. In the WGP, we used seven regression methods. RR^40^, BL^41^, EBL^34^, wBSR^42^, and Bayes C^43^ are linear models, whereas RKHS^44^ and RF^45^ are nonlinear models. We used R package *rrBLUP* version 4.3 for MLM, RR, and RKHS, and *randomForest* version 4.6–7 for RF. For BL, EBL, wBSR, and Bayes C, we used C language programs developed by Onogi *et al.*^61^ that were based on variational Bayesian algorithms. For MLM, an additive kinship matrix was used as the covariance between lines due to the polygenic effect, and six principal components (PCs) were included as the fixed effects. This number of PCs was selected because this number successfully detected previously identified QTLs such as *fw9.1*^35^ and *Lycopersicum Invertase 5*^37^. For other regression methods, fixed effects such as the result of Bayesian clustering were not used because few differences were observed in the results. The linear regression models using significant associations in the GWAS as explanatory variables were built with R function ‘lm’. If two or more significant associations were detected, variable selection was conducted using Akaike’s Information Criterion (AIC). The calculation of AIC values and variable selection was conducted using the R function ‘step’.

### Simulation

The tomato genome in this simulation study was represented by the linkage map from Shirasawa *et al.*^28^, with a bin size of 0.1 cM. The number of recombinations on each chromosome was determined using a random variable drawn from a Poisson distribution. For each chromosome, the lambda parameter of the Poisson distribution (i.e. the expected value of the random variable) was set as the length of the linkage map (in Morgan) estimated by Shirasawa *et al.*^28^. The position of each recombination in a chromosome was sampled from a uniform distribution by ignoring the recombination interference. For the construction of genotype data for the simulated genome, the genotype of each marker was determined on the basis of the haplotype of the nearest bin in the simulated genome. In the recurrent selection, the population size was kept constant at *n* = 96. Namely, the number of progenies from each cross in the first generation was 24 (96/4 crosses), whereas the number was 12 (96/8 crosses) in the subsequent generations (Fig. 3a). Via simulation, 96 inbred lines derived from each F_1_ variety were produced by six generations of inbreeding. To simulate inbred lines derived from each generation of the recurrent selection, one inbred line was produced from each individual in the parental population. A total of 5 independent simulations were performed. All analyses for the simulations were written and conducted in R (http://www.R-project.org/).

## Additional Information

**How to cite this article**: Yamamoto, E. *et al.* A simulation-based breeding design that uses whole-genome prediction in tomato. *Sci. Rep.* **6**, 19454; doi: 10.1038/srep19454 (2016).

## Supplementary Material

Supporting Information

Supplementary Data S1

Supplementary Data S2

Supplementary Data S3

## Figures and Tables

**Table 1:** List of traits analysed in this study.

Trait	Abbreviation	Trait category	*h*^2^	Details
Percentage of fruit set (%)	PF	Yield	0.300	Percentage of flowers that reached fruit set in a plant
Total fruit weight (g/plant)	TFW	Yield	0.507	Total fruit weight per plant
Average fruit weight (g)	AFW	Yield	0.538	Average weight of all fruits from a plant
Percentage of marketable fruits (%)	PMF	Yield	0.401	Percentage of fruits of 100 g or more, without physiological disorders, in a plant
Total marketable fruit weight (g/plant)	TMFW	Yield	0.449	Total marketable fruit weight per plant
Average marketable fruit weight (g)	AMFW	Yield	0.469	Average weight of marketable fruits in a plant
Soluble solids content (˚Brix)	SSC	Quality	0.600	Degree of Brix measured by saccharimeter (average of 4 marketable fruits per plant)
Pericarp colour	PCol	Quality	1.000	Colourless (pink tomato) and yellow (red tomato) pericarp counted as 0 and 1, respectively
Style scar	SS	Quality	0.492	Size of style scar on ripened fruit was scored based on the length of major axis. 0: <4 mm, 1: 4 ~ 10 mm, 2: >10 mm
Percentage of blossom-end rot fruits (%)	PBF	Physiological disorder of fruit	0.389	Percentage of blossom-end rot fruits in a plant
Percentage of irregular-shaped fruits (%)	PIF	Physiological disorder of fruit	0.478	Percentage of irregular-shaped fruits in a plant
Percentage of cracked fruits (%)	PCF	Physiological disorder of fruit	0.338	Percentage of cracked fruits in a plant
Percentage of small fruits (%)	PSF	Physiological disorder of fruit	0.372	Percentage of fruits less than 100 g in a plant
Leaf length (mm)	LL	Others	0.492	Length of a leaf under the first truss
Leaf width (mm)	LW	Others	0.464	Width of a leaf under the first truss
Stem width (mm)	SW	Others	0.377	Width of a stem at the position of the first truss
Height to the first truss (cm)	H1T	Others	0.370	Height of the first truss from ground
Number of flowers	NFlo	Others	0.382	Number of flowers after defloration (maximum number of flowers is 6 per truss)
Days to flowering	DTF	Others	0.106	Number of days from seeding to first flower
Number of leaves under the first truss	NL1T	Others	0.389	Number of true leaves under the first truss

**Table 2:** Linear regression model that uses significant associations in genome-wide association.

Trait	NSM*1	NUM*2	*r*^2^
Average fruit weight	2	1	0.047
Average marketable fruit weight	1	1	0.067
Soluble solids content	3	3	0.535
Pericarp colour	3	3	0.648
Style scar	1	1	0.220
Percentage of blossom-end rot fruits	1	1	0.322
Percentage of irregular-shaped fruits	4	3	0.368
Percentage of cracked fruits	6	4	0.378
Leaf length	3	3	0.386
Leaf width	3	3	0.199
Stem width	1	1	0.021
Height to the first truss	1	1	0.020
Number of flowers	1	1	0.075
Days to flowering	3	3	0.443
Number of leaves under the first truss	2	1	0.137

^*1^NSM, Number of significant markers in the GWAS.

^*2^NUM, Number of markers used in the linear regression models. The variable selection was conducted using Akaike’s Information Criterion. See Methods for the details.

**Table 3:** Accuracy of genomic estimated breeding values (GEBVs) in traits evaluated in this study.

Trait	RR	BL	EBL	wBSR	Bayes C	RKHS	RF
Percentage of fruit set	0.238	0.244	0.220	***0.319***	0.290	0.256	0.207
Total fruit weight	0.590	0.591	0.576	0.602	***0.606***	0.599	0.472
Average fruit weight	0.461	0.424	***0.461***	0.450	0.450	0.455	0.302
Percentage of marketable fruits	0.199	0.206	0.133	0.238	***0.256***	0.225	0.017
Total marketable fruit weight	0.403	0.381	0.377	0.400	***0.414***	0.413	0.118
Average marketable fruit weight	0.437	0.387	***0.482***	0.429	0.420	0.408	0.221
Soluble solids content	0.772	0.768	***0.807***	0.779	0.778	0.771	0.679
Pericarp colour	0.482	0.371	0.456	0.387	0.465	***0.514***	0.498
Style scar	***0.513***	0.493	0.496	0.505	0.511	0.495	0.508
Percentage of blossom-end rot fruits	−0.064	0.113	0.015	0.030	0.153	***0.206***	0.012
Percentage of irregular-shaped fruits	0.454	0.439	0.406	0.448	***0.465***	0.427	0.413
Percentage of cracked fruits	−0.018	0.034	−0.240	0.095	0.022	0.117	***0.422***
Percentage of small fruits	−0.048	0.131	−0.030	0.018	***0.156***	−0.103	−0.049
Leaf length	0.361	0.366	0.244	0.307	***0.384***	0.346	0.365
Leaf width	0.282	0.307	0.302	0.328	***0.335***	0.305	0.213
Stem width	0.336	***0.353***	0.337	0.340	0.347	0.342	0.258
Height to the first truss	0.397	0.409	0.381	***0.415***	0.399	0.355	0.394
Number of flowers	0.332	0.367	0.350	0.323	***0.376***	0.331	0.343
Days to flowering	0.576	0.584	0.581	0.580	0.591	***0.703***	0.653
Number of leaves under the first truss	0.285	0.275	0.212	0.311	0.304	***0.326***	0.276

Accuracy was evaluated as a Pearson’s correlation coefficient between phenotypic values and GEBVs from leave-one-out cross validation.

Bold italics indicate the highest value in the same trait.

RR, Ridge regression; BL, Bayesian Lasso; EBL, Extended Bayesian Lasso; wBSR, Weighted Bayesian shrinkage regression; RKHS, Reproducing kernel Hilbert space regression; RF, Random forest.
