# Supplementary material for: A simulation-based breeding design that uses whole-genome prediction in tomato
Source: Sci Rep. 2016 Jan 20;6:19454. doi: 10.1038/srep19454 (PMC4726135; doi:10.1038/srep19454)
Supplement: Supporting Information [file srep19454-s1.pdf]

Supplementary Information

A simulation-based breeding design that uses whole-genome prediction in tomato

Eiji Yamamoto[1], Hiroshi Matsunaga[1], Akio Onogi[2], Hiromi Kajiya-Kanegae[2], Mai Minamikawa[2], Akinori Suzuki[2], Kenta Shirasawa[3], Hideki Hirakawa[3], Tsukasa Nunome[1], Hirotaka Yamaguchi[1], Koji Miyatake[1], Akio Ohyama[4], Hiroyoshi Iwata[2] & Hiroyuki Fukuoka[1]

[1]NARO Institute of Vegetable and Tea Science (NIVTS), 360 Kusawa, Ano, Tsu, Mie 514-2392, Japan. [2]Department of Agricultural and Environmental Biology, Graduate School of Agricultural and Life Sciences, The University of Tokyo, 1-1-1 Yayoi, Bunkyo-Ku, Tokyo 113-8657, Japan. [3]Kazusa DNA Research Institute, 2-6-7 Kazusa-kamatari, Kisarazu, Chiba 292-0818, Japan. [4]NARO Institute of Vegetable and Tea Science (NIVTS), 3-1-1 Kannondai, Tsukuba, Ibaraki 305-8666, Japan. Correspondence and requests for materials should be addressed to E.Y. (email: yame@affrc.go.jp)

Supplementary Tables S1–S3
Supplementary Figures S1–S3
Supplementary Methods
Supplementary Note
References

Supplementary Table S1 List of tomato varieties used in the present study

Variety	Year of development	Hierarchical clustering	Bayesian clustering	
			K1	*K2*
SL1	2009	2	0.82	0.18
SL2	1985	1	0.34	0.66
SL3	1998	2	0.82	0.18
SL4	1975	4	0.00	1.00
SL5	1985	1	0.17	0.83
SL6	2005	3	0.83	0.17
SL7	NA	1	0.42	0.58
SL8	1971	4	0.00	1.00
SL9	2000	3	0.86	0.14
SL10	1955	4	0.00	1.00
SL11	1983	1	0.10	0.90
SL12	1990	3	1.00	0.00
SL13	NA	3	1.00	0.00
SL14	1991	3	0.97	0.03
SL15	1976	4	0.00	1.00
SL16	2003	3	1.00	0.00
SL17	1976	4	0.00	1.00
SL18	2000	3	1.00	0.00
SL20	1976	4	0.00	1.00
SL21	1952	4	0.00	1.00
SL22	1991	2	0.75	0.25
SL23	1996	3	1.00	0.00
SL24	1987	3	0.89	0.11
SL25	1979	4	0.09	0.91
SL26	2005	2	0.82	0.18
SL27	1994	1	0.72	0.28
SL28	1980	1	0.52	0.48
SL29	1979	4	0.07	0.93
SL30	2008	3	1.00	0.00
SL31	NA	1	0.43	0.57
SL32	2007	2	0.64	0.36
SL33	1967	4	0.00	1.00
SL34	2009	3	1.00	0.00
SL35	1977	4	0.16	0.84
SL36	1980	4	0.13	0.87
SL37	1973	4	0.00	1.00
SL38	1989	3	0.93	0.07
SL39	2001	3	1.00	0.00
SL40	1992	1	0.35	0.66
SL41	1996	3	1.00	0.00
SL42	1997	3	1.00	0.00
SL43	1985	1	0.32	0.68
SL44	1982	4	0.00	1.00
SL45	2002	2	0.82	0.18
SL46	1970	1	0.51	0.49
SL47	NA	1	0.55	0.45
SL48	1999	2	0.72	0.28
SL49	1989	1	0.44	0.56
SL50	1970	4	0.00	1.00

Supplementary Table S1 (*Continued*)

Variety	Year of development	Hierarchical clustering	Bayesian clustering	
			K1	*K2*
SL51	1971	4	0.01	0.99
SL52	1998	2	0.69	0.31
SL53	1981	4	0.11	0.89
SL54	2009	3	1.00	0.00
SL55	1980	1	0.58	0.42
SL56	1976	1	0.43	0.57
SL57	1984	1	0.38	0.62
SL58	NA	4	0.00	1.00
SL59	2009	2	0.85	0.16
SL60	NA	1	0.36	0.64
SL61	2009	2	0.85	0.15
SL62	1952	4	0.00	1.00
SL63	1980	4	0.06	0.94
SL64	1984	4	0.23	0.77
SL65	1983	1	0.41	0.59
SL66	NA	3	0.83	0.17
SL67	1993	2	0.79	0.21
SL68	2008	3	0.98	0.02
SL69	1973	4	0.02	0.98
SL70	2000	4	0.21	0.80
SL71	1981	4	0.09	0.91
SL72	NA	3	0.97	0.03
SL73	NA	1	0.50	0.50
SL74	2009	3	1.00	0.00
SL75	2009	3	1.00	0.00
SL76	2004	3	1.00	0.00
SL77	1982	1	0.36	0.64
SL79	1982	3	1.00	0.00
SL80	1994	3	1.00	0.00
SL81	1974	1	0.58	0.42
SL82	1981	1	0.42	0.58
SL83	1986	1	0.54	0.46
SL84	2007	2	0.74	0.26
SL85	1994	3	1.00	0.00
SL86	1999	2	0.60	0.40
SL87	2009	2	0.88	0.12
SL88	2000	2	0.64	0.36
SL89	1981	4	0.00	1.00
SL90	2008	3	0.97	0.03
SL91	1986	1	0.69	0.31
SL92	NA	2	0.60	0.40
SL93	2009	2	0.89	0.11
SL94	1997	2	0.75	0.25
SL95	1985	1	0.44	0.56
SL96	1981	1	0.30	0.70
SL102	NA	1	0.11	0.89
SL103	NA	4	0.00	1.00

Supplementary Table S2 List of the significant associations detected by the genome-wide association study

Trait	SNP ID	Chr	Position (bp)	Position (cM)	r^2	MLM		EBL					
						Effect	$-\log_{10}P$	θ	Effect	SD	τ^2	δ^2	η^2
AFW	AX-95768470	9	4894274	51.94	0.01	-24.703	2.716	0.1	-17.74	1.69	2.22	222.98	0.03
								1	-23.92	1.28	0.69	674.21	0.00
								5	-28.50	1.03	0.31	1357.09	0.00
AFW	AX-107553846	9	5015774	52.72	0.05	-14.161	3.503	0.0001	-9.23	2.18	17.95	2.46	21.86
								0.001	-9.67	2.11	15.12	15.83	2.86
AMFW	AX-107553846	9	5015774	52.72	0.07	-14.925	4.410	0.0001	-10.49	2.03	11.63	2.49	14.02
								0.001	-10.97	1.96	9.82	15.95	1.84
								0.01	-12.02	1.72	6.12	68.90	0.27
								0.1	-13.03	1.40	3.41	223.93	0.05
								1	-13.83	1.07	1.74	675.51	0.01
								5	-13.70	0.89	1.23	1359.07	0.00
SSC	AX-95816341	2	37533453	70.70	0.29	0.131	1.188	0.0001	0.15	0.03	9.77	2.26	12.95
								0.001	0.17	0.03	7.51	14.61	1.54
SSC	AX-107530294	5	626924	3.83	0.29	-0.085	0.467	0.0001	-0.24	0.03	3.81	2.26	5.04
SSC	AX-95792472	9	2623609	34.38	0.07	-0.271	5.579	0.0001	-0.23	0.04	4.27	2.26	5.66
								0.001	-0.23	0.04	4.29	14.61	0.88
								0.01	-0.23	0.03	3.30	66.42	0.15
								0.1	-0.25	0.03	1.69	222.49	0.02
								1	-0.27	0.02	0.80	674.44	0.00
								5	-0.27	0.02	0.51	1357.11	0.00
PCol	AX-107528977	1	70228553	49.28	0.32	-0.124	3.009	0.1	-0.14	0.01	0.88	220.68	0.01
								1	-0.13	0.01	0.53	670.25	0.00
								5	-0.12	0.00	0.40	1352.94	0.00
PCol	AX-95802300	1	71269940	51.30	0.58	-0.273	9.198	0.0001	-0.33	0.01	0.32	2.52	0.38
								0.001	-0.33	0.01	0.30	15.87	0.06
								0.01	-0.32	0.01	0.23	68.32	0.01
PCol	AX-95782963	1	75814558	59.34	0.22	0.126	3.749	0.1	0.10	0.01	1.75	220.68	0.02
								1	0.10	0.01	0.90	670.25	0.00
								5	0.10	0.00	0.55	1352.94	0.00
SS	AX-107526519	9	1937367	29.36	0.22	0.279	3.491	0.0001	0.20	0.04	6.28	2.56	7.36
								0.001	0.20	0.04	6.01	16.30	1.11
PBF	AX-107540607	4	62766820	133.53	0.32	-3.493	5.800	-	-	-	-	-	-
PIF	AX-107529487	1	85767014	132.77	0.10	2.085	2.195	1	1.72	0.18	3.12	674.13	0.01
								5	1.63	0.15	2.39	1355.61	0.01
PIF	AX-107526307	3	508999	9.97	0.14	3.443	4.285	0.0001	2.61	0.36	5.64	2.55	6.64
								0.001	2.62	0.35	5.24	16.28	0.97
								0.01	2.72	0.31	3.72	69.82	0.16
								0.1	2.47	0.24	2.74	224.31	0.04
								1	1.83	0.18	2.77	674.13	0.01
PIF	AX-107532554	4	1786073	23.46	0.17	3.468	3.616	0.1	1.93	0.24	4.53	224.31	0.06
								1	2.06	0.18	2.19	674.13	0.01
PIF	AX-107544746	9	1874011	28.56	0.00	1.737	1.533	5	2.26	0.24	1.25	1355.61	0.00
PCF	AX-95814346	2	46569227	131.42	0.20	-0.674	3.757	0.01	-0.40	0.05	4.84	68.39	0.21
PCF	AX-95775213	3	8107799	51.09	0.15	-0.549	3.021	0.1	-0.31	0.05	5.03	222.17	0.07
								1	-0.41	0.03	1.52	672.13	0.01
								5	-0.45	0.03	0.81	1354.87	0.00
PCF	AX-107545745	4	4722129	45.06	0.00	-0.458	2.900	1	-0.20	0.04	5.76	672.13	0.03

Supplementary Table S2 (*Continued*)

Trait	SNP ID	Chr	Position (bp)	Position (cM)	r^2	MLM		EBL					
						Effect	$-\log_{10}P$	θ	Effect	SD	τ^2	δ^2	η^2
PCF	AX-95773156	5	63169277	119.67	0.14	-0.604	4.205	0.0001	-0.32	0.08	11.23	2.48	13.56
								0.001	-0.34	0.08	9.65	15.80	1.83
								0.01	-0.27	0.06	10.63	68.39	0.46
PCF	AX-95789137	5	63705710	124.20	0.00	0.506	2.671	1	0.33	0.04	2.28	672.13	0.01
								5	0.46	0.03	0.77	1354.87	0.00
PCF	AX-105346901	7	58641570	42.46	0.02	0.544	3.092	0.1	0.32	0.05	4.64	222.17	0.06
LL	AX-95774169	2	30666575	18.40	0.07	2.475	3.836	0.0001	1.34	0.36	22.20	2.58	25.81
								0.001	1.47	0.35	17.09	16.27	3.14
								0.01	1.75	0.32	9.30	69.69	0.40
								0.1	1.61	0.24	6.42	224.41	0.09
								1	2.07	0.21	2.78	675.58	0.01
								5	2.09	0.17	1.79	1357.70	0.00
LL	AX-107526715	2	46747730	132.16	0.19	-3.723	3.326	0.1	-2.15	0.29	3.61	224.41	0.05
LL	AX-107530056	3	49391068	59.87	0.24	-3.514	3.198	0.1	-2.07	0.26	3.88	224.41	0.05
LW	AX-107544905	1	80430590	100.56	0.09	3.277	2.512	1	2.28	0.18	1.57	674.51	0.01
								5	2.78	0.15	0.72	1357.70	0.00
LW	AX-95774169	2	30666575	18.40	0.03	3.834	2.247	0.1	1.61	0.24	6.03	223.47	0.08
								1	1.90	0.17	2.24	674.51	0.01
								5	1.93	0.14	1.49	1357.70	0.00
LW	AX-107537130	2	31577517	22.85	0.08	3.020	2.324	1	1.79	0.18	2.52	674.51	0.01
								5	1.54	0.15	2.31	1357.70	0.01
SW	AX-107544905	1	80430590	100.56	0.02	0.852	1.709	1	0.50	0.06	4.28	675.17	0.02
								5	0.73	0.05	1.42	1357.81	0.00
H1T	AX-107541971	1	86566633	137.79	0.02	-4.788	2.095	5	-2.84	0.27	2.23	1356.42	0.00
NFlo	AX-107552308	2	48487492	151.84	0.07	1.719	3.333	0.1	1.07	0.10	2.45	224.15	0.03
DTF	AX-107529487	1	85767014	132.77	0.12	0.570	1.313	1	0.35	0.07	12.45	674.89	0.05
DTF	AX-107530056	3	49391068	59.87	0.29	0.926	1.851	1	1.16	0.08	1.23	674.89	0.01
								5	0.80	0.07	1.93	1357.28	0.00
DTF	AX-105347391	6	36178958	45.78	0.00	0.651	1.735	1	0.47	0.10	7.11	674.89	0.03
NL1T	AX-107528273	3	50678874	59.87	0.00	0.461	2.343	1	0.45	0.04	1.39	675.13	0.01
								5	0.59	0.03	0.54	1357.45	0.00
NL1T	AX-95814127	5	2899948	37.22	0.14	0.345	2.220	0.0001	0.23	0.07	17.89	2.61	20.55

PF, percentage of fruit set; TFW, total fruit weight; AFW, average fruit weight; PMF, percentage of marketable fruits; TMFW, total marketable fruit weight; AMFW, average marketable fruit weight; SSC, soluble solids content; PCol, pericarp color; SS, style scar; PBF, percentage of blossom-end rot fruits; PIF, percentage of irregular shaped fruits; PCF, percentage of cracked fruits; PSF, percentage of small fruits; LL, leaf length; LW, leaf width; SW, stem width; H1T, height to the first truss; NFlo, number of flowers; DTF, days to flowering; NL1T, number of leaves under the first truss. See Table 1 for the details.

Supplementary Table S3 Summary of the design of a single-nucleotide polymorphism (SNP) genotyping array

SL2.40ch	Number of candidate SNPs	Q > 30	Number of polymorphic reads > 2	Number of reads on the SNP site < 500	Number of SNPs provided for 50K SNP array	Number of SNPs provided for the genetic analysis
1	1,350,865	1,020,741	430,057	401,966	5,929	1,801
2	990,576	766,610	333,923	318,994	5,062	1,452
3	940,293	703,851	283,441	260,973	4,477	1,220
4	949,622	729,925	323,682	307,347	5,333	1,821
5	827,602	631,362	276,057	257,613	4,010	774
6	1,015,221	832,195	461,013	444,392	4,087	866
7	843,100	643,182	276,804	256,564	3,093	984
8	801,849	607,382	258,392	239,352	3,582	1,097
9	1,364,857	1,171,846	758,392	734,549	4,023	1,474
10	863,897	669,628	307,091	284,550	3,681	1,690
11	830,820	652,913	323,610	306,922	4,612	2,273
12	891,116	703,253	345,836	322,588	4,023	1,330
Total	11,669,818	9,132,888	4,378,298	4,135,810	51,912	16,782

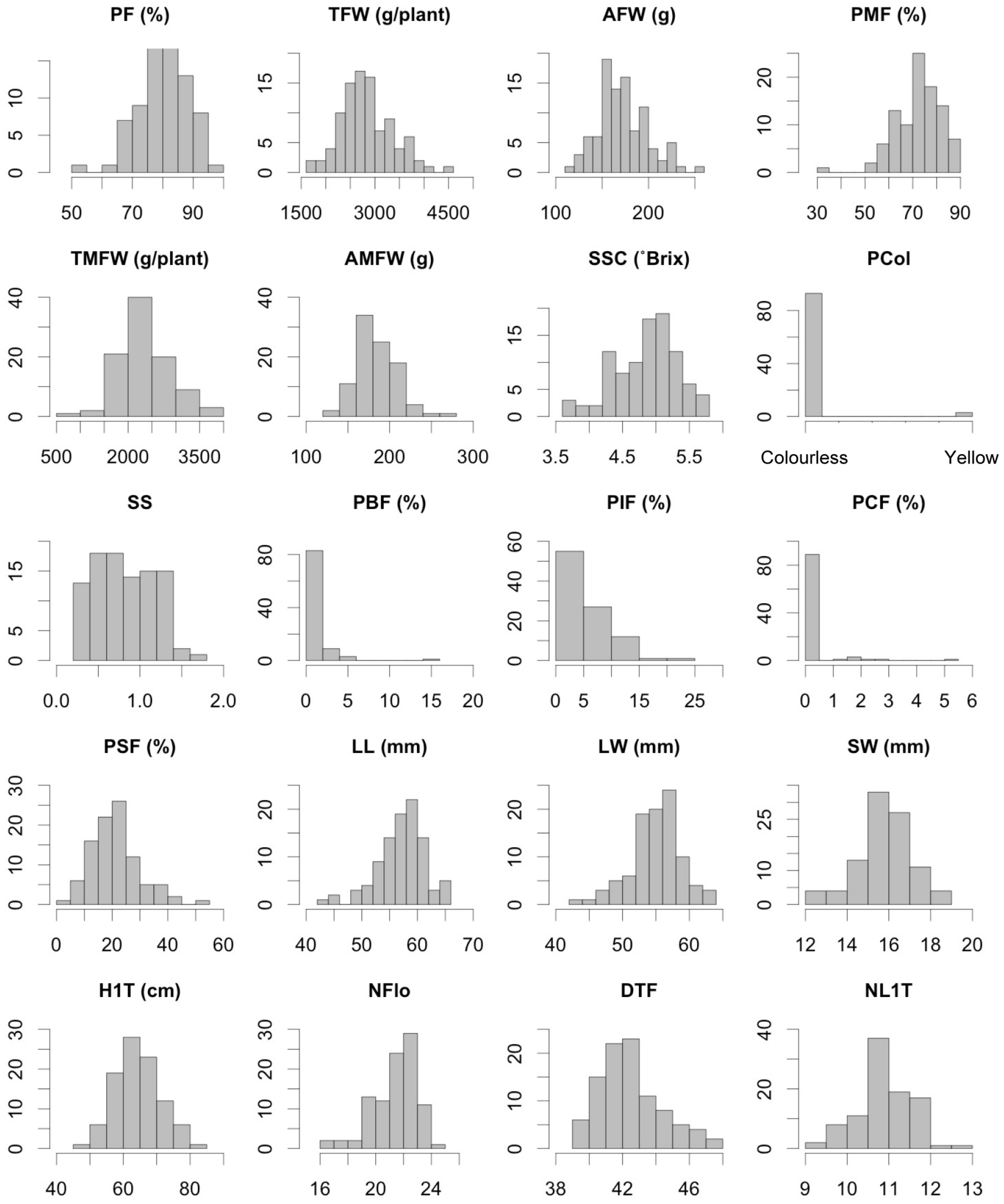

Supplementary Figure S1 Phenotypic distributions of the 96 tomato varieties used in the present study. PF, percentage of fruit set; TFW, total fruit weight; AFW, average fruit weight; PMF, percentage of marketable fruits; TMFW, total marketable fruit weight; AMFW, average marketable fruit weight; SSC, soluble solids content; PCol, pericarp colour; SS, style scar; PBF, percentage of blossom-end rot fruits; PIF, percentage of irregular-shaped fruits; PCF, percentage of cracked fruits; PSF, percentage of small fruits; LL, leaf length; LW, leaf width; SW, stem width; H1T, height to the first truss; NFlo, number of flowers; DTF, days to flowering; NL1T, number of leaves under the first truss. See Table 1 for details.

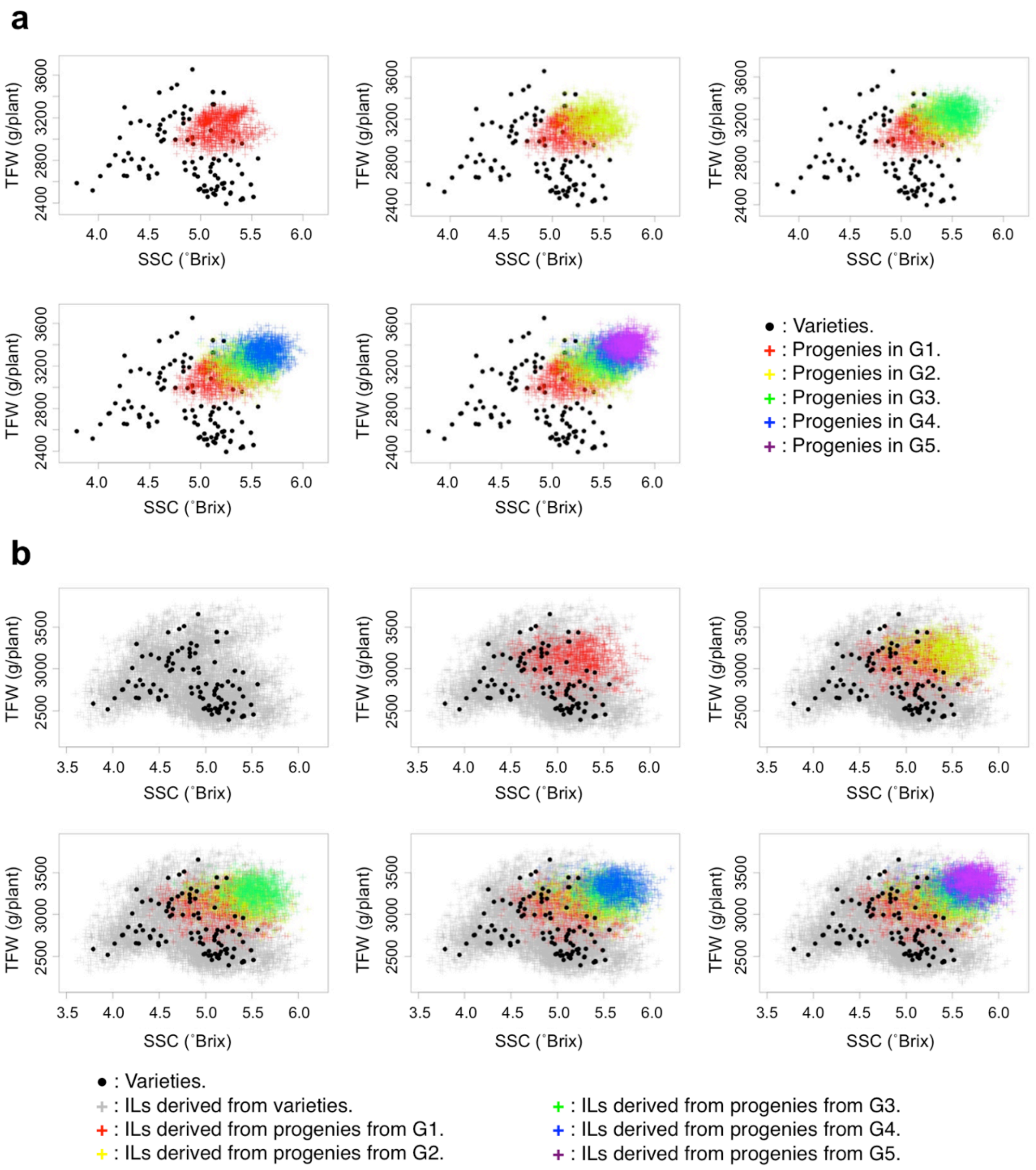

Supplementary Figure S2 Changes in the genomic estimated breeding values (GEBVs) in the simulations of the recurrent genomic selection, specifically, distributions of the GEBVs of total fruit weight (TFW) and soluble solids content (SSC). Black circles and coloured crosses indicate the GEBVs of the 96 varieties and the simulated populations, respectively. G1 to G5 indicate the progenies derived from one to five cycles of recurrent selection for which the breeding strategy is shown in Fig. 3. (a) Distribution of GEBVs of breeding population during recurrent genomic selection. (b) Distribution of GEBVs of inbred lines (ILs) derived from breeding population during recurrent genomic selection (Fig. 3b and Supplementary Fig. S2a).

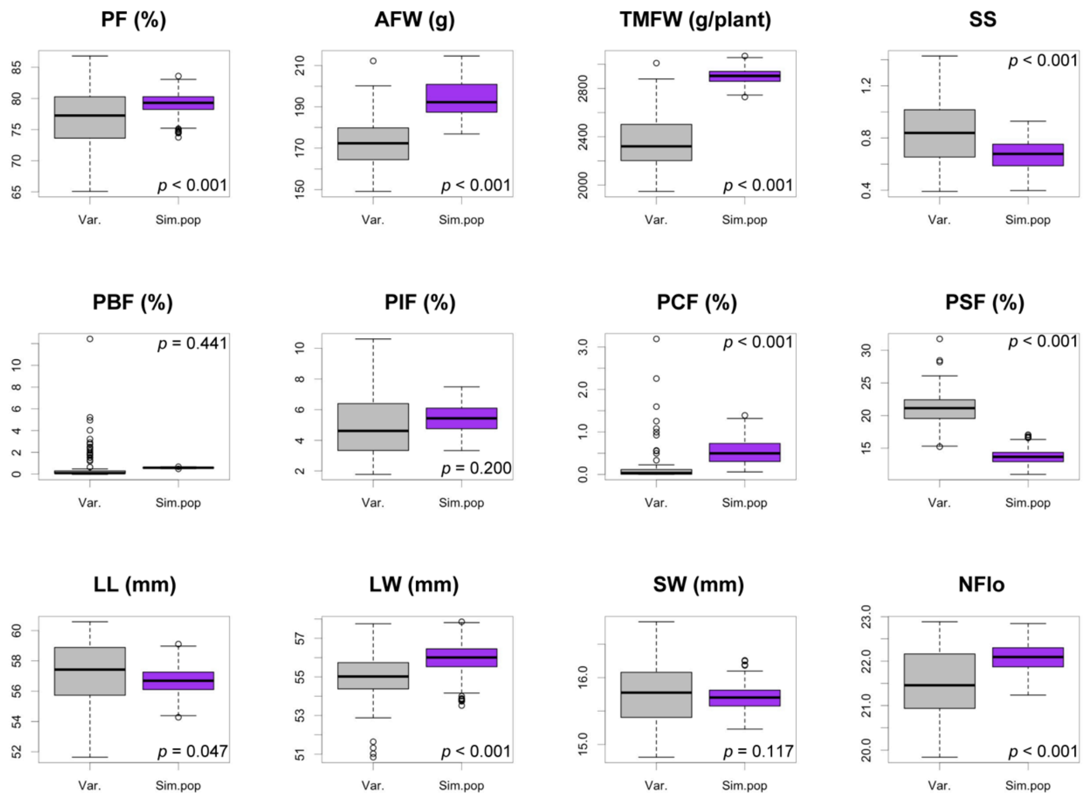

Supplementary Figure S3 Influence of the selection for total fruit yield and soluble solids content on other traits. Boxplots for the genomic estimated breeding values in the fifth generation of the simulated population (G5 in Fig. 3b and Supplementary Fig. S2a). 'Var.' and 'Sim.pop.' at the bottom of each panel indicate the 96 varieties and the simulated population, respectively. Statistical analysis was performed using Welch's *t*-test. PF, percentage of fruit set; AFW, average fruit weight; TMFW, total marketable fruit weight; SS, style scar; PBF, percentage of blossom-end rot fruits; PIF, percentage of irregular-shaped fruits; PCF, percentage of cracked fruits; PSF, percentage of small fruits; LL, leaf length; LW, leaf width; SW, stem width; NFlo, number of flowers.

Supplementary Methods

Single-nucleotide polymorphism (SNP) discovery and design of a SNP genotyping array

DNA samples from the 96 tomato F_1 varieties were sequenced on a HiSeq2000 (Illumina, San Diego, CA, USA) lane using 100-base-pair end sequencing. A total of 173 Gb sequences were obtained, which corresponds to a 1.90× depth for the genome of each variety (DDBJ Sequence Read Archive Submission DRA003755). Obtained reads were aligned against the tomato reference genome (Tomato Genome Consortium, 2012) (release SL2.40) by using CLC Genomic Workbench version 6.5 (CLC bio, a QIAGEN Company, Aarhus, Denmark). Repetitive sequences on the tomato reference genome were excluded based on the annotation dataset ITAG2.30. A total of 11,669,818 putative SNP sites were discovered (Supplementary Table S3). The following criteria were used for the selection of SNPs:

1. The average of quality value Q was > 30. Q is defined by $Q = -log_{10}(P)$, where P is the probability of an incorrect base call.
2. The number of reads that showed polymorphism against the reference genome was > 2.
3. The number of reads obtained at the putative SNP site was < 500.

Criteria 1 and 2 were used to exclude SNPs with low reliability. Criterion 3 was used to avoid the selection of repetitive genomic regions that were undefined in ITAG2.30. By using these criteria, the number of candidate SNPs was narrowed down to 4,135,810 (Supplementary Table S3). The linkage map position (cM) of these SNPs was estimated by local polynomial regression fitting by using linkage map information from Shirasawa *et al.* (2010) as the predictors. According to the estimated linkage map position, the tomato genome was divided into 1-cM blocks, and an average 35 SNPs were selected from each block. The selected SNPs were evenly distributed on the tomato genome according to their linkage map positions. These procedures were important aspects of an objective analysis of linkage disequilibrium (LD) and breeding simulation. Specifically, because the tomato genome has a large pericentromeric region (Tomato Genome Consortium, 2012), selection of SNPs on the basis of physical positions or according to random selection will result in the selection of many SNPs on centromeric regions, which will affect the interpretation of the LD analysis and make it

difficult to determine the recombination position in a simulation study.

A total of 51,912 SNPs were selected for analysis with the Axiom myDesign Genotyping Array (Affymetrix Co, Ltd., Santa Clara, CA, USA). In this genotyping assay, 35,247 SNPs were polymorphic in the varieties used in the present study. From these polymorphic SNPs, the SNPs with minor allele frequency < 0.05 and a rate of missing genotype > 0.05 were excluded. Finally, 16,782 SNPs were provided for further analysis (Fig. 1a, Supplementary Table S3).

Regression methods

MLM: This method assumes the following model (Yu *et al.*, 2006):

$$y = X\beta + Zg + \varepsilon \quad (2)$$

where y is a vector of phenotypes; X is a matrix of fixed effects including the grand mean, SNPs, and other variables; β is a vector of fixed effects; and Z is an incidence matrix mapping each observed phenotype to one individual. The variable g models the genetic background of each line as a random effect with $Var[g] = K\sigma^2$. K is the kinship matrix inferred from the genotypes. The residual variance is $Var[\varepsilon] = I\sigma^2$. An additive kinship matrix was used as the covariance between the lines due to a polygenic effect. Six principal components (PCs) were included as fixed effects. We used function 'GWAS' in the R package *rrBLUP* (Endelman, 2011).

RR: This method was examined by Meuwisssen *et al.* (2001), which assumes the model

$$y = 1_n\mu + \sum_i \boldsymbol{W}q_i + e \quad (3)$$

where 1_n is a vector of ones; μ is the mean; W is a matrix that contains genotypes code as 0, 1, or 2; q_i is the effect of each SNP; and e is a vector of random normal deviates with variance σ_e^2. The elements in W in each column j have an amount $2p_j$ (where p_j is the minor allele frequency of marker j) subtracted from the genotype code to achieve that the sum of coefficients in each column is zero. The SNP effects are treated as random and summed over all segments. The genetic variance explained by the SNP effects is given by $\boldsymbol{WW'}\sigma_q^2$ and the residual variance is $I\sigma_e^2$, and the variance–covariance matrix among observations is $var(y) = \boldsymbol{WW'}\sigma_q^2 + I\sigma_e^2$. The variance for each SNP can be assumed equal. Habier *et al.* (2007) showed that RR is equivalent to Genomic best linear unbiased prediction (gBLUP). In gBLUP, the model is given by

$$y = 1_n\mu + \boldsymbol{Z}g + e \quad (4)$$

where $\boldsymbol{Z}$ is a design matrix allocating records to genetic values and g is a vector of the additive genetic effects of markers. The variance of g is $\boldsymbol{G}\sigma_g^2$ where $\boldsymbol{G}$ is the genomic relationship matrix and σ_g^2 is the genetic variance for this model. The variance of y in this model is given by $\boldsymbol{ZGZ}'\sigma_q^2 + I\sigma_e^2$. We used the R package *rrBLUP* version 4.2 (Endelman, 2011) to fit the RR-BLUP model.

BL: In BL, the following linear model was used (Park & Casella, 2008):

$$y_i = \sum_{p=1}^{P} x_{ip}\beta_p + \varepsilon_i \quad (5)$$

where y_i is a phenotypic value of individual i, x_{ip} is a genotype of marker p of individual i, β_p is a effect of marker *p*, and ε_i is a residual for the individual *i* with $\varepsilon_i \sim N(0, \sigma_e^2)$. In BL, each regression parameter β_p is assumed to be normally distributed around zero with its own variance σ_p^2, and the degree of shrinkage is locus-specific by the variance σ_p^2 across loci. According to Li and Sillanpää (2012) and Onogi *et al.* (2015), β_p was assumed to follow

$$\beta_p \sim N\left(0, {}^{1}\!/_{\tau_0^2\tau_p^2}\right) \quad (6)$$

where τ_p^2 determines the magnitude of shrinkage for β_p, and $1/\tau_0^2$ is the residual variance, respectively. Then, τ_p^2 was assumed to follow a prior distribution

$$\tau_p^2 \sim Inv - G\left(1, {}^{\lambda^2}\!/_{2}\right) \quad (7)$$

where $Inv - G$ indicates the inverse Gamma distribution and λ^2 is a regularisation parameter that defines the distribution of τ_p^2. The prior distribution of λ^2 was

$$\lambda^2 \sim G(1, \varpi) \quad (8)$$

where ϖ is the rate parameter. In BL, ϖ was the hyperparameter, and five values of ϖ were tested: 0.001, 0.01, 0.1, 1, and 5. Parameters were estimated by using variational Bayesian approaches (VBA). A nested five-fold cross-validation (CV) was performed for each cycle of LOOCV (Table 3) to determine the optimal hyperparameter value that showed the least mean square error.

EBL: EBL is the extension of BL that separates the regularisation parameter λ^2 into a

shrinkage factor for the overall model sparsity and a shrinkage factor for individual markers (Mutshinda & Sillanpää, 2010). This approach is intended to assign different magnitudes of shrinkage to individual marker effects. In EBL, a prior distribution of τ_p^2 was described as follows;

$$\tau_p^2 \sim Inv - G\left(1, {}^{\delta^2\eta_p^2}/_2\right) \quad (9)$$

where δ^2 is the shrinkage factor for all markers and η_p^2 is the shrinkage factor unique to marker p. A prior distribution for δ^2 was $\delta^2 \sim G(1,1)$, and for η_p^2 was $\eta_p^2 \sim G(1,\theta)$ where the rate parameter θ is the hyperparameter for EBL. Six values of θ were tested: 0.0001, 0.001, 0.01, 0.1, 1, and 5. As was the case for BL, parameters were estimated by using VBA (Mutshinda & Sillanpää, 2010) and a nested five-fold CV was performed to determine the optimal hyperparameter.

wBSR: A linear model for wBSR is

$$y_i = \sum_{p=1}^{P} \gamma_p x_{ip} \beta_p + \varepsilon_i \quad (10)$$

where γ_p is the indicator variable that determines whether the marker effect is included in the regression model ($\gamma_p = 1$) or not ($\gamma_p = 0$) (Hayashi & Iwata, 2010). A prior distribution was

$$\gamma_p \sim Bernoulli(\pi) \quad (11)$$

If $\gamma_p = 1$, β_p was assumed to follow

$$\beta_p \sim N(0, \sigma_p^2) \quad (12)$$

then the prior for σ_p^2 was

$$\sigma_p^2 \sim \chi^{-2}(v, S) \quad (13)$$

where χ^{-2} indicates a scaled inverse chi-square distribution, v is a degree-of-freedom, and S is a scale parameter. In this study, we used 1 for v and S, thus π was the only hyperparameter adjusted. Five values of π were tested: 0.01, 0.1, 0.2, 0.5, and 1. Parameters were estimated by using VBA (Hayashi & Iwata, 2013) and a nested five-fold CV was performed to determine the optimal hyperparameter.

Bayes C: In Bayes C, the general statistical model is the same as in BL and EBL, whereas the mechanism for the shrinkage of marker effects is similar to that of wBSR

(Habier *et al.*, 2011). The prior expression for β_p in Bayes C was described as follows;

$$\beta_p = \begin{cases} 0 & if\ \rho_p = 0 \\ \sim N(0, \sigma^2) & if\ \rho_p = 1 \end{cases} \quad (14)$$

where ρ_p is the indicator variable that determines whether the marker effect is included in the regression model ($\rho_p = 1$) or not ($\rho_p = 0$), with the prior distribution

$$\rho_p = Bernoulli(\pi) \quad (15)$$

In BL, EBL, and wBSR, each SNP has its own variance, whereas all SNP effects have a common variance σ^2 in Bayes C. A prior distribution of σ^2 was described as follows:

$$\sigma^2 \sim \chi^{-2}(v, S) \quad (16)$$

The sets of hyperparameters tested were the same as in wBSR. VBA (Carbonetto & Stephens, 2012) was used for the parameter estimations and a nested five-fold CV was performed to determine the optimal hyperparameter.

RKHS: In linear regressions, phenotypes relate to marker genotype linearly. However, non-additive genetic effects, such as dominance and epistatic effects, render the relations non-linear. Kernel functions are often used to capture this non-additive relationship. The relationship model can be written as

$$K_{ij} = \langle G_{i\cdot}, G_{j\cdot} \rangle \quad (17)$$

where the angle brackets denote the inner (or dot) product between genotype *i* and *j*. In RKHS, the following kernel was used:

$$K_{ij} = exp\left[-\left(D_{ij}/\theta\right)^2\right] \quad (18)$$

where

$$D_{ij} = \left[(1/4M) \sum_{k=1}^{M} (G_{ik} - G_{jk})^2\right]^{1/2} \quad (19)$$

is the Euclidean distance between marker genotypes *i* and *j*, normalised to interval [0, 1]. M is the number of markers and the parameter θ is a scale parameter that influences how quickly the genetic covariance decays with distance. RKHS is equivalent to replacing the genomic relationship matrix $\boldsymbol{G}$ with the Gaussian kernel matrix $\boldsymbol{K}$ where each element is based on K_{ij}. RKHS is described by Gianola *et al.* (2006), Gianola and van Kaam (2008), and de los Campos *et al.* (2009). RKHS was performed using the R package *rrBLUP* version 4.2 (Endelman, 2011) with the default setting.

RF: RF is an ensemble learning method that uses a combination of decision trees, each generated from a subset of variables (markers in genomic prediction) selected by bootstrap (Breiman, 2001). RF avoids over-fitting by using stochastic perturbation (bootstrap) and averaging the outputs of the decision trees (Hastie *et al.*, 2009). RF was performed using the R package *randomForest* versions 4.6-7. All parameters were set to their default values, that is, the number of variables tried at each split $m_{try} = p/3$, number of trees = 500, and minimum node size = 5.

Supplementary Note

Validity assessment of use of averaged phenotypic values over a range of years

The phenotypic values of agronomic traits are often subject to genotype by environment (G×E) effects, which often disturb genetic analyses such as a genome-wide association study (GWAS) or whole-genome prediction (WGP) model. In the present study, we used phenotypic values averaged over four years for GWAS (Fig. 2) and WGP (Table 3), which enabled us to ignore G×E effects. We chose this approach for two reasons: (i) because only one plant was grown for each variety, each year, it is difficult to analyse G×E effects using standard statistical methods, and (ii) under such conditions, the use of averaged values is the most conservative method. However, a validity assessment for the use of the averaged phenotypic value is needed in order to estimate the reliability of the result.

We performed the following analysis to estimate the G×E effects in the present study. The analysis was composed of four experiments (Supplementary Fig. S4). In each experiment, the phenotypic values from four years were divided into two groups: phenotypic values from one year as test data and phenotypic values from the other three years as training data. The phenotypic values from three years were averaged and used for construction of a WGP model. Then the genomic estimated breeding values (GEBVs) based on the WGP model were compared with the phenotypic values from the test data. GEBVs of each trait were calculated using the statistical method that showed the highest predictability in Table 3 of the main text. The accuracy of each WGP model for the test data was evaluated as a Pearson's correlation coefficient between the phenotypic values and the GEBVs (Supplementary Fig. S4). Strong G×E effects would be revealed by the presence of low correlation coefficients.

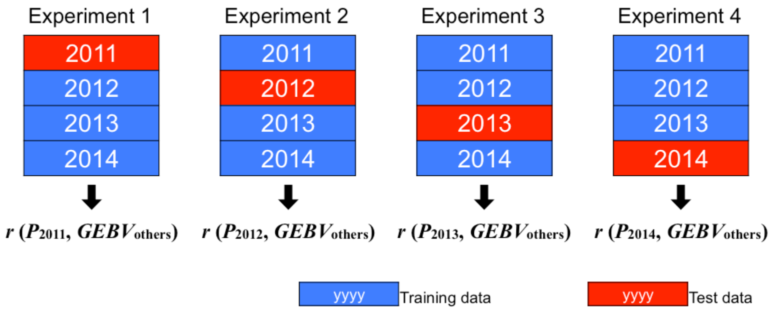

Supplementary Figure S4 Schematic representation of the analysis to assess the validity of using averaged phenotypic values over the years.

Most of the traits showed similar correlation coefficients across the four experiments (Supplementary Table S4). In particular, total fruit weight and soluble solids content, traits that were an important focus of the main text, showed high correlation coefficients for all experiments (Supplementary Table S4). This finding indicates that the G×E effect is small for these traits in the present study.

Regarding traits categorized as 'physiological disorder of fruit', such as percentage of blossom-end rot fruits, percentage of cracked fruits, and percentage of small fruits (Table 1), several experiments showed extremely low correlation coefficients (Supplementary Table S4). Because the trait physiological disorder of fruit is easily subjected to changes in environmental conditions compared with other traits, this result is reasonable. However, in the experiments that showed extremely low correlation coefficients, the frequency of occurrence of physiological disorder was extremely low in the test data. In other words, plant growth was stable and did not differ between years. Therefore, we concluded that the G×E effect in the present study was not strong and that it is reasonable to use the averaged phenotypic values over the years for GWAS and WGP.

Supplementary Table S4 Validity assessment of use of averaged phenotypic values over the years

Trait	Experiment 1	Experiment 2	Experiment 3	Experiment 4
Percentage of fruit set	0.372	0.325	0.048	0.119
Total fruit weight	0.593	0.535	0.553	0.570
Average fruit weight	0.407	0.568	0.543	0.532
Percentage of marketable fruits	0.013	0.461	0.284	0.323
Total marketable fruit weight	0.403	0.474	0.390	0.505
Average marketable fruit weight	0.409	0.561	0.576	0.445
Soluble solids content	0.632	0.663	0.787	0.705
Style scar	0.453	0.439	0.526	0.432
Percentage of blossom-end rot fruits	0.444	-0.003	0.317	NA
Percentage of irregular-shaped fruits	0.517	0.402	0.389	0.383
Percentage of cracked fruits	0.202	-0.034	NA[*1]	NA
Percentage of small fruits	-0.103	0.363	0.278	0.297
Leaf length	0.297	0.552	0.322	NA
Leaf width	0.419	0.200	0.347	NA
Stem width	0.286	0.221	0.438	NA
Height to the first truss	0.481	0.583	0.404	NA
Number of flowers	0.304	0.187	0.444	0.325
Days to flowering	0.289	0.272	NA	0.447
Number of leaves under the first truss	0.139	0.354	0.187	NA

Values are Pearson's correlation coefficients between phenotypic values from a year and genomic estimated breeding values (GEBVs) based on the other three years (Supplementary Fig. S4).

NA, not analysed because phenotypic record for that year was not available.

[*1] Correlation coefficient of percentage of cracked fruits in experiment 3 was incalculable because a very small number of varieties showed a non-zero phenotype.

References

- Breiman, L. Random forests. *Machine Learning* **45,** 5-32 (2001).
- Carbonetto, P. & Stephens, M. Scalable variational inference for Bayesian variable selection in regression, and its accuracy in genetic association studies. *Bayesian Analysis* **7,** 73-108 (2012).
- de Los Campos, G., Gianola, D. & Rosa, G.J.M. Reproducing kernel Hilbert spaces regression: a general framework for genetic evaluation. *J. Anim. Sci.* **87,** 188 (2009).
- Endelman, J.B. Ridge regression and other kernels for genomic selection with R package rrBLUP. *Plant Genome* **4,** 250-255 (2011).
- Gianola, D., Fernando, R.L., & Stella, A. Genomic-assisted prediction of genetic value with semiparametric procedures. *Genetics* **173,** 1761-1776 (2006).
- Gianola, D. & van Kaam, J.B. Reproducing kernel Hilbert spaces regression methods for genomic assisted prediction of quantitative traits. *Genetics* **178,** 2289-2303 (2008).
- Habier, D., Fernando, R.L. & Dekkers, J.C. The impact of genetic relationship information on genome-assisted breeding values. *Genetics* **177,** 2389-2397 (2007)
- Habier, D., Fernando, R.L., Kizilkaya, K. & Garrick, D.J. Extension of the bayesian alphabet for genomic selection. *BMC Bioinformatics* **12,** 186 (2011).
- Hastie, T., Tibshirani, R. & Friedman, J. The elements of statistical learning. *Springer, New York* (2009).
- Hayashi, T. & Iwata, H. EM algorithm for Bayesian estimates of genomic breeding values. *BMC Genetics* **11,** 3 (2010).
- Hayashi, T. & Iwata, H. A Bayesian method and its variational approximation for prediction of genomic breeding values in multiple traits. *BMC Bioinformatics* **14,** 31 (2013).
- Li, Z. & Sillanpää, M.J. Estimation of quantitative trait locus effects with epistasis by variational Bayes algorithms. *Genetics* **190,** 231-249 (2012).
- Meuwissen, T.H.E., Hayes, B.J. & Goddard, M.E. Prediction of total genetic value using genome-wide dense marker maps. *Genetics* **157,** 1819-1829 (2001).
- Mutshinda, C.M. & Sillanpää, M.J. Extended Bayesian LASSO for multiple quantitative trait loci mapping and unobserved phenotype prediction. *Genetics* **186,** 1067-1075 (2010).

- Onogi, A. *et al*. Exploring the areas of applicability of whole-genome prediction methods for Asian rice (*Oryza sativa* L.). *Theor. Appl. Genet.* **128,** 41-53 (2015).
- Park, T. & Casella, G. The Bayesian LASSO. *J. Am. Stat. Assoc.* **103,** 681-686 (2008).
- Shirasawa, K. *et al*. An interspecific linkage map of SSR and intronic polymorphism markers in tomato. *Theor. Appl. Genet.* **121,** 731–739 (2010).
- Tomato Genome Consortium. The tomato genome sequence provides insights into fleshy fruit evolution. *Nature* **485,** 635–641 (2012).
- Yu *et al*. A unified mixed-model method for association mapping that accounts for multiple levels of relatedness. *Nat Genet* **38,** 203-208 (2006).
